# “Steering” Toward Complete Left Atrial Appendage Closure

**DOI:** 10.1016/j.jscai.2024.101358

**Published:** 2024-03-22

**Authors:** Safi U. Khan, Sachin S. Goel

**Affiliations:** Department of Cardiovascular Medicine, Houston Methodist DeBakey Heart and Vascular Center, Houston, Texas

**Keywords:** Left atrial appendage closure, peri-device leak, steerable delivery sheaths

Left atrial appendage closure (LAAC) has emerged as an important alternative for stroke prevention in patients with nonvalvular atrial fibrillation who are at increased risk for stroke but not good candidates for long-term anticoagulation.^1^^,^^2^ However, there are safety concerns related to device-related thrombus and peri-device leak (PDL), both of which are associated with increased stroke risk.^1^ Anatomical variations of the left atrial appendage (LAA) heighten the complexity of achieving desired outcomes. For instance, achieving coaxial alignment between the delivery sheath and the LAA neck is paramount, a task sometimes complicated by challenging LAA morphologies.^1^ Delivery sheaths used routinely are not deflectable or steerable. The advent of steerable delivery sheaths (SDS), offering adjustable distal end angles from 0° to 120°, provides a potential solution to navigate these anatomical challenges.^3^^,^^4^ Small series and case reports have highlighted the safety and potential for improved outcomes using SDS in LAAC.^3^, ^4^, ^5^ Chang et al^4^ assessed the feasibility of FlexCath Steerable Sheath (Medtronic) in implanting Watchman (Boston Scientific) and Amulet (Abbott) devices, achieving successful implantation with lower PDL rates through optimal coaxial alignment.

In this issue of *JSCAI,* Amabile et al^6^ provide further insights by exploring the utilization of SDS for LAAC with the Amulet device in patients with significantly enlarged left atria on computed tomography (LA volume index >90 mL/m^2^). This single-center study analyzed the procedural outcomes of 47 patients—22 using the SDS and 25 with a standard delivery sheath. The primary end point was procedural success (defined as the exclusion of LAA without any device-related complications, no leak >5 mm on intraprocedural color Doppler transesophageal echocardiogram, and no procedure-related complications), and secondary end points were the presence of disc/lobe misalignment, patent LAA, and PDL on postprocedural CT scan. Both groups exhibited similar demographics, comorbidities, and procedural characteristics in the analysis. The study demonstrated comparable procedural success and an absolute 46% reduction in residual patent LAA, a 48% decrease in PDL, and a 27% reduction in the risk of off-axis device position with SDS compared with the standard sheath. However, these findings should be approached with caution due to potential selection bias stemming from narrow inclusion criteria, the significant reduction—from 387 to 47—of eligible patients, and the possibility of attrition bias, as only 41 patients received a follow-up CT scan.

With these considerations in view, it is essential to contextualize these results within the broader landscape of the safety challenges of LAAC, especially regarding PDL. In the PROTECT-AF trial (WATCHMAN Left Atrial Appendage System for Embolic PROTECTion in Patients With Atrial Fibrillation), 40.9% of patients had PDL at 45 days, decreasing to 32.1% at 1 year, with leaks >3 mm affecting 13.3% initially and 11.8% at 1 year.^7^ The Amulet IDE trial reported 37% PDL at 45 days with the Amulet device, compared with 54% with the Watchman device; leaks >3 mm were detected in 10% and 25% of patients, respectively.^8^ The NCDR LAAO registry noted PDL in 26.6% of patients at 45 days, linking small leaks (<5 mm) to an increased risk of ischemic stroke and transient ischemic attack over 1 year.^9^ A large meta-analysis including 61,666 patients who underwent LAAC found that transesophageal echocardiography-detected PDL after LAAC was associated with an increased risk of thromboembolic adverse events. This risk had a positive graded association across cut-offs of PDL size (0, 1, 3, and 5 mm).^10^ These data highlight the significance of minimizing PDL in LAAC procedures, and the advances in new occluder devices and the introduction of SDS promise improvements in sealing the LAA, aiming for better procedural outcomes.^1^

Furthermore, a substantial proportion of the patients undergoing mitral transcatheter edge-to-edge repair (mTEER) have atrial fibrillation and face a high bleeding risk.^3^ Combining mTEER with LAAC is practical once access to the left atrium is achieved through a transeptal procedure. Typically, LAAC is performed using an inferoposterior transseptal puncture, whereas MitraClip (Abbott) procedures rely on a high and posterior puncture approach. The SDS technology could enable LAAC device placement through a higher puncture site, similar to that used for MitraClip, enhancing procedural feasibility and efficiency.^7^

In conclusion, this study by Amabile et al^6^ adds to the growing experience of SDS in successful device deployment for LAAC. This preliminary evidence paves the way for expanded research and clinical integration. Future studies should pursue broader, multicenter trials with extended follow-up and diverse populations to refine generalizability, focusing on different LAA anatomical variations and comorbidity profiles. Advancements in guidance tools and imaging, including 3D imaging and advanced navigation, are essential for navigating complex LAA anatomies. Collaborative efforts among specialists would be instrumental in integrating these innovations, steering toward safer, more effective LAAC strategies.
